# Effect of multi-epitope derived from HIV-1 on REM sleep deprivation-induced spatial memory impairment with respect to the level of immune factors in mice

**DOI:** 10.22038/IJBMS.2022.61175.13536

**Published:** 2022-02

**Authors:** Roya Lahimgarzadeh, Salar Vaseghi, Mohammad Nasehi, Fatemeh Rouhollah

**Affiliations:** 1 Department of Cellular and Molecular Biology, Faculty of Advanced Science and Technology, Tehran Medical Sciences, Islamic Azad University, Tehran, Iran; 2 Medicinal Plants Research Center, Institute of Medicinal Plants, ACECR, Karaj, Iran; 3 Department of Cognitive Neuroscience, Institute for Cognitive Science Studies (ICSS), Tehran, Iran; 4 Cognitive and Neuroscience Research Center (CNRC), Amir-Almomenin Hospital, Tehran Medical Sciences, Islamic Azad University, Tehran, Iran

**Keywords:** Epitopes, HIV-1, Immunologic factors, Sleep deprivation, Spatial memory

## Abstract

**Objective(s):**

Sleep deprivation (SD) has a negative impact on cognitive functions including learning and memory. Many studies have shown that rapid-eye-movement (REM) SD also disrupts memory performance. In this study, we aimed to investigate the effect of multi-epitope Gag-Pol-Env-Tat derived from Human immunodeficiency virus 1 (HIV-1) on REM SD-induced spatial memory impairment with respect to the levels of interleukin-4 (IL-4), interleukin-17 (IL-17), interferon-gamma (IFN-γ), immunoglobulin G1 (IgG1), immunoglobulin G2a (IgG2a), and lymphocyte proliferation in NMRI mice. We used multi-epitope Gag-Pol-Env-Tat derived from HIV-1 because Gag-Pol-Env-Tat immunogen sequence is one of the most sensitive immunogen sequences of HIV-1 that can significantly augment cellular and humoral immune systems, leading to the improvement of cognitive functions.

**Materials and Methods:**

Morris water maze apparatus was used to assess spatial memory, and multi-platform apparatus was used to induce RSD for 24 hr. Multi-epitope derived from HIV-1 was subcutaneously injected at the dose of 20 µgr/ml, once and fourteen days before RSD.

**Results:**

RSD impaired spatial memory and injection of multi-epitope derived from HIV-1 reversed this effect. RSD decreased IL-4, IgG1, and IgG2a levels, while multi-epitope derived from HIV-1 reversed these effects. Multi-epitope derived from HIV-1 also increased lymphocyte proliferation and decreased IL-17 levels in both control and RSD mice.

**Conclusion:**

Multi-epitope derived from HIV-1 may improve memory performance via induction of anti-inflammatory immune response.

## Introduction

Evidence shows the crucial role of sleep in regulating cognitive functions (1). A large number of studies have shown the important role of sleep in modulating learning and memory processes (2-4). Furthermore, recent reports have shown the impairment effect of total sleep deprivation (TSD) or REM (rapid eye movement) sleep deprivation (RSD) on memory function (5, 6). Importantly, sleep and the immune system have interactions with each other. Sleep is an important predictor of immunity since sleep deprivation (SD) affects the development of the immune response and increases susceptibility to contracting an infection (7). SD negatively affects the central nervous system (CNS) and the immune system (8). Previous reports have revealed that RSD plays a role in regulating changes in the immune system and increases the production of pro-inflammatory cytokines and cells, which participate in the innate immune response (9). Yehuda *et al*. have also shown that the level of interleukin-17 (IL-17) is increased following RSD (9). In addition, chronic SD significantly increases the expression level of interleukin-1β (IL-1β) and tumor necrosis factor-α (TNF-α) in the brain (10). Studies have also shown that multiple infectious diseases associated with sleep disorders, especially infectious agents such as viruses, bacteria, and parasites, infect the CNS and based on the immune response against pathogen infection or its direct effect on CNS, induce sleep disturbances (11, 12). 

Poor sleep is a common complaint of individuals with human immunodeficiency virus (HIV) and acquired immune deficiency syndrome (AIDS) (13). Human immunodeficiency virus-1 (HIV-1) may directly induce sleep disturbances, while its exact mechanism is unknown (14). Although, it has been suggested that the high risk of poor sleep may be related to antiretroviral medication (15). Individuals with HIV show impaired or poor cognitive functions in many areas such as attention, memory, concentration, psychomotor, and executive functioning (16). It has been shown that poorer self-reported sleep quality is related to poorer self-reported cognitive functions in individuals with HIV (17). Furthermore, the level of interferon-gamma (IFN-γ) and IL-17 is high in individuals with HIV (18). In general, pro-inflammatory markers are increased in individuals with HIV (19). However, some immunogen design strategies using HIV-1 proteins can improve cognitive functions and immune system activity, or induce therapeutic effects on immune system-related disorders such as acquired immunodeficiency syndrome (AIDS) (20). For example, it has been shown that the HIV-1-derived p24-gp41 immunogen sequence significantly augments immune responses in Balb/c mice (21). A recent study has also reported that multi-epitope p24-Nef derived from HIV-1 also increases the expression of interleukin-4 (IL-4), which may stimulate the humoral immune response (22). In this study, we used the Gag-Pol-Env-Tat immunogen sequence because this sequence is one of the most sensitive immunogen sequences of HIV-1, which can significantly augment cellular and humoral immune systems.

According to the mentioned findings, the goal of this study is to evaluate the effect of multi-epitope Gag-Pol-Env-Tat derived from HIV-1 on RSD-induced spatial memory impairment with respect to the level of IL-4, IL-17, IFN-γ, immunoglobulin G1 (IgG1), immunoglobulin G2a (IgG2a), and lymphocyte proliferation in mice. Gag, Pol, and Env are structural proteins of HIV-1, and Tat is an essential regulatory protein of this virus. 

## Materials and Methods

***Animals***

Sixty-four NMRI male mice, 6 to 8 weeks old, purchased from Pasteur Institute of Iran, were used in this research. All mice were kept under stable temperature (22+2 °C) and standard light/dark cycle (12/12 hr, lights on at 8 am to 8 pm). Free access to food and water for mice was provided. All experiments were carried out during the light phase (between 8 am and 4 pm). Our experimental protocol was approved by the Research and Ethics Committee of the School of Advanced Technologies in Medicine, Tehran University of Medical Sciences, and was done in accordance with the National Institutes of Health Guide for the Care and Use of Laboratory Animals (NIH publications No. 80–23).

***Preparation of the multi-epitope Gag-Pol-Env-Tat***

Refolded multi-epitope recombinant protein, which was obtained from the plasmid pET23a-HIVtop4, Gag158-186, Pol150-190, ENV296-323, ENV577-610, Tat1-20, and Tat44-61 in strains of *E. coli* BL21 with a purity of 95%, a molecular weight of 23 KDa, and a concentration of 20 µg, was purchased from Pasteur Institute, Tehran, Iran (23).

***Experimental groups and procedure***

The mice were classified into two main groups including REM sleep-deprived (RSD for 24 hr) and control. Each main group consisted of four groups: mice receiving PBS, mice receiving adjuvant montanide (Adj), mice receiving multi-epitope Gag-Pol-Env-Tat without adjuvant montanide (M), and mice receiving multi-epitope Gag-Pol-Env-Tat with adjuvant montanide (M+Adj). Each group consisted of eight male mice. The multi-epitope Gag-Pol-Env-Tat was subcutaneously injected in the amount of 20 µgr/ml (24) only once and before RSD. 14 days later, 24 hr RSD was performed. After RSD, spatial learning and memory were assessed. Seven days later, blood sampling was carried out from the eye retro-orbital area and then, the mice were killed by cervical dislocation and the spleen was extracted. 

***REM SD apparatus***

Multiple-platform apparatus (BorjSanatAzma Co, Tehran, Iran) was applied to induce RSD for 24 hr. All mice were placed in a water tank (90*50*50 cm) which included several circular platforms with 7 cm diameter. The surface of the platforms was 2 cm above the water surface. During the experiment, all mice were free to move through the tank, from one platform to another platform. When the REM stage started, the mice fell into the water and woke up, due to muscles relaxing during the REM phase. This process induced RSD. The temperature of the water was standard and monitored during the test, the light-dark cycle (12/12 h) was also considered and food and water were provided for mice (5). 

***Morris water maze***

*The apparatus*

Fourteen days after injections, the mice were trained in the Morris Water Maze (MWM) apparatus. MWM is an authentic apparatus to evaluate spatial performance in rodents (25). MWM is a circular black tank (150 cm in diameter and 60 cm depth) filled with 20±2 °C water to a depth of 30 cm. Various stable visual signs in shape and size were placed on the room walls around the tank. The tank was divided into four equal quadrants. For each quadrant, there was one starting site: north (N), south (S), west (W), and east (E). The north-west quadrant was considered the “target quadrant” and the hidden platform (10 cm in diameter) was submerged 1 cm into the water in the center of the target quadrant. During the experiment, the swimming of each rat was recorded by a camera located above the tank. The camera was also connected to a computer. A video tracking system (Etho-Vision XTv 8.5; Noldus Information Technology, Wageningen, the Netherlands) was used to assess three parameters in the training session: escape latency (time needed to find the hidden platform), traveled distance (path length traveled to find the hidden platform), and swimming speed (swimming velocity during training); and two parameters in the probe session: escape latency and traveled distance in the target quadrant (25).

*Behavioral procedure*

Each training session for each mouse consisted of eight trials with four different starting positions. Each of the starting positions was equally distributed around the perimeter of the maze. Each trial was started by placing each mouse in one of the quadrants. The swimming period was 60 sec. Distal spatial cues (different in shape and size) guided mice to find the hidden platform. After finding the platform, mice were allowed to remain on the platform for 20 sec to memorize the situation. Then, they were placed in a cage for 20 sec until the start of the next trial. If a mouse did not locate the hidden platform during this period, it was manually guided to the platform by the researcher. The spent time, the traveled distance, and the swimming speed during each training were measured. A probe trial was done 24 hr after the trainings to assess spatial memory retrieval. Before performing the probe trial, the hidden platform was removed from the tank. The probe trial consisted of a 60 sec free-swimming period, with measuring the time spent and the traveled distance only in the target quadrant for measuring spatial memory retrieval. For non-spatial visible test evaluation, the platform was elevated 2 cm above the water and covered with a piece of aluminum foil in the center of the northeast quadrant. This test was performed after the probe trial. This procedure can provide information on the possible non-specific effects involving motor, visual, or motivational abilities unrelated to learning and memory (26). 

***Extraction and culture of mouse spleen cell***

Seven days after behavioral evaluation, the spleen of mice was isolated in sterile conditions to measure IL-4, IL-17, and IFN-γ, and proliferation of lymphocyte cells, and placed in PBS containing 5% FCS (Sigma-Aldrich - Germany). In each group, with the help of lysis buffer comprising Tris-base 2 % and NH4CL 8/0 % (pH 7.5), red blood cells were destroyed. The cell suspension was obtained with 20 × 106 cells. The medium contained RPMI-1640 with 5% FCS, 50 mM -2 mercaptoethanol, 4 mmol -L-glutamine, 1 mM sodium pyruvate, 100 micrograms per ml streptomycin, and 100 IU/ml penicillin (Sigma-Aldrich - Germany). 

***Evaluation of lymphocyte proliferation with BrdU method***

Cell suspension (100 μl / well) was placed in ELISA plates, 20 micrograms per milliliter recombinant poly-epitope and 5 micrograms per milliliter Phyto Hemaglutinin (PHA) per well were added in case and positive control groups, respectively. No antigen was added to the negative control wells. 100 ml solution of 5-bromo - 2 deoxyribose - uridine (as the cursor) was added to all wells and after 18 hr of re-culture, 300 g of samples were centrifuged for 15 min. 100 ml antibodies against BrdU and 100 ml solution of tetra-methyl benzidine were subsequently added into each well, and the reaction was stopped with 100 ml of 2N sulfuric acid, and the results were calculated based on absorbance at 450 nm stimulation index SI. 

***Evaluation of IL-4, IL-17, and IFN-γ***

Evaluation of IL-4, IL-17, and IFN-γ was done using R and D Systems Quantikine ELISA kits according to the manufacturer’s protocol. This assay was performed in spleen cell suspensions of 4 × 106 cells rich in culture medium RPMI-1640. Evaluation of IL-4 and IFN-γ is in order to assess humoral and cellular immune responses, respectively. The expression level of IL-17 was measured as an inflammatory factor. 

***Evaluation of IgG1 and IgG2a***

Humoral immune response was performed by ELISA, to evaluate IgG1 and IgG2a antibodies. The coating stage of the multi-epitope recombinant Gag-Pol-Env-Tat was performed with the bottom of ELISA plate, 100 microliters per well of poly-epitope recombinant incubation conditions for 24 hr at 4 ° C, and in the darkness. Washing was carried out with buffer PBS, containing 0.5% Tween-20 for 2 hr at 37 °C, and after blocking, incubation, and washing stages, each well was supplemented with 100 ml of diluted serum samples at concentrations of 1 to 10 to 1 in 1280. Serum samples were prepared by the ophthalmic blood collection from the Retro orbital area. After the collection, blood samples were centrifuged at 37 °C for 90 min, with 8000 rpm for ten minutes. After washing several times, the amount of 100 ml 1/10000 concentration of mice IgG specific HRP binding antibody was added to each well (Sigma-Aldrich - Germany) and incubated for 70 to 90 min at 37 °C. After washing (5 times) with the amount of 100 ml, TMB substrate was added in the darkness. The reaction was stopped with 2N sulfuric acid and absorbance at the wavelength of 450 nm was measured by ELISA reader. 

***Statistical analysis***

Data were analyzed using the SPSS (V.22) software. At first, the normality of data was checked using the Kolmogorov-Smirnov test (data not shown). Then, One-way ANOVA and *post hoc* Tukey’s were used to analyze the results. The statistical significance level was considered as less than 0.05 and the measurement results were expressed as mean±SD. 

## Results

***Results of spatial learning***

**Figure 1:**
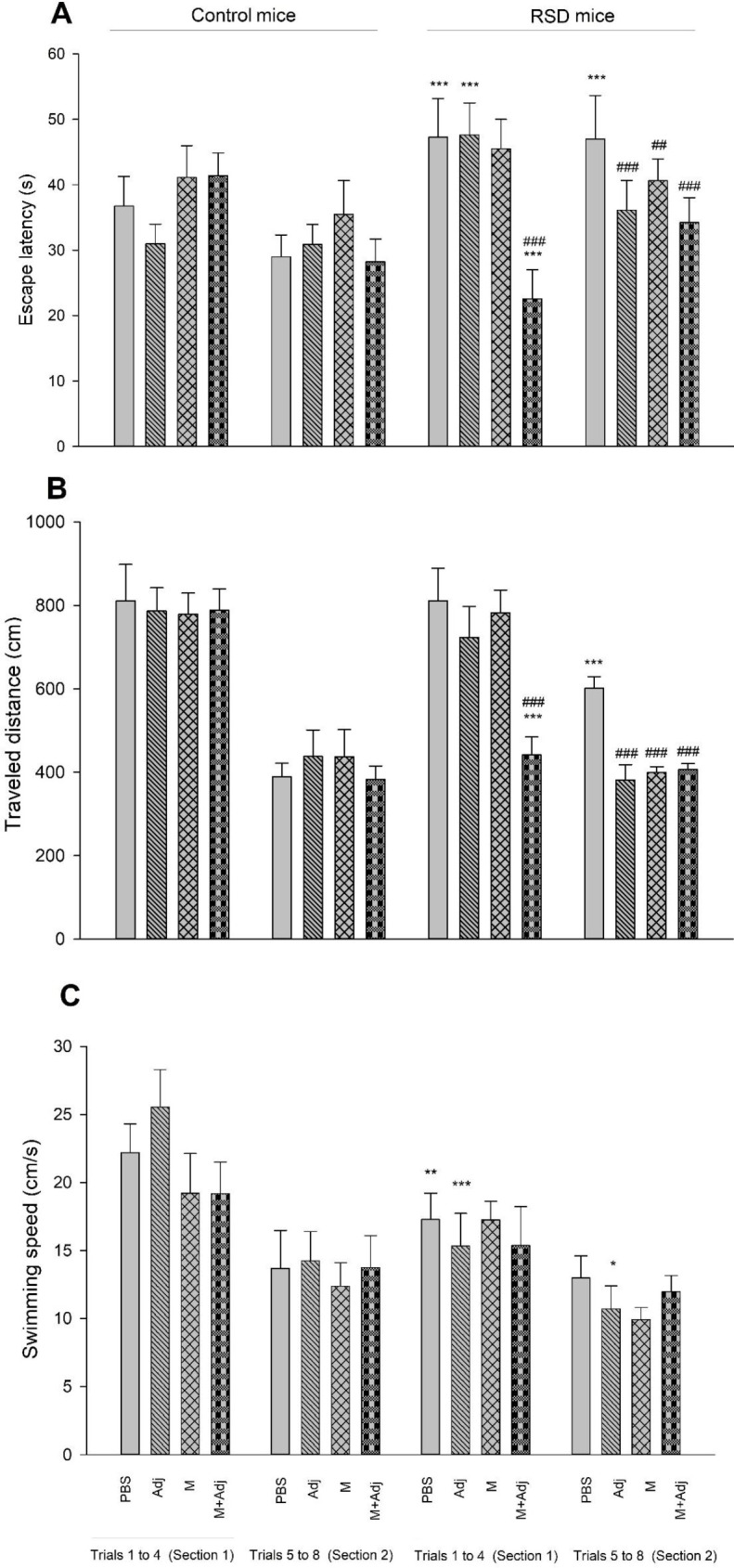
Effect of REM sleep deprivation (RSD), adjuvant montanide (Adj), multi-epitope Gag-Pol-Env-Tat derived from HIV-1 (M), and multi-epitope Gag-Pol-Env-Tat derived from HIV-1 + adjuvant montanide (M+Adj) on spatial learning (PBS as control). Panel A shows the time spent to find the hidden platform, Panel B shows the traveled distance to find the hidden platform, and Panel C shows the swimming speed during each 60 sec trial. Section 1 is the mean data of trials 1 to 4, and section 2 is the mean data of trials 5 to 8. (***P<0.001, **P<0.01, and *P<0.05 in comparison with the related group of control mice, ###*P*<0.001, ##*P*<0.01, and #*P*<0.05 in comparison with RSD mice receiving PBS; n = 8)

*Escape latency-* The results of one-way ANOVA showed that there is a significant difference between groups in the first four trials (average data for each mouse) (F_7,56_ = 20.98, *P*<0.001). *Post hoc* Tukey’s also showed that RSD mice receiving PBS (*P*<0.001) or Adj (*P*<0.001) used more time to find the hidden platform in the target quadrant in the first four trials than their related groups of control mice, indicating spatial learning impairment. However, mice receiving M+Adj used less time to find the hidden platform in the target quadrant in the first four trials than their related group of control mice (*P*<0.001), and also RSD mice receiving PBS (*P*<0.001), indicating spatial learning improvement. Furthermore, the results of one-way ANOVA showed that there is a significant difference between groups in the second four trials (average data for each mouse) (F_7,56_ = 16.94, *P*<0.001). *Post hoc* Tukey’s also showed that RSD mice receiving PBS (*P*<0.001) used more time to find the hidden platform in the target quadrant in the second four trials than their related group of control mice, indicating spatial learning impairment. While, all groups of RSD mice (Adj: *P*<0.001, M: *P*<0.01, and M+Adj: *P*<0.001) used less time, indicating spatial learning improvement. As the results showed, RSD impaired spatial learning and multi-epitope Gag-Pol-Env-Tat derived from HIV-1 improved spatial learning impairment in RSD mice (Figure 1, Panel A).

*Traveled distance-* The results of one-way ANOVA showed that there is a significant difference between groups in the first four trials (average data for each mouse) (F_7,56_ = 30.32, *P*<0.001). *Post hoc* Tukey’s also showed that RSD mice receiving M+Adj used less time to find the hidden platform in the target quadrant in the first four trials than their related group of control mice (*P*<0.001), and also RSD mice receiving PBS (*P*<0.001), indicating spatial learning improvement. Furthermore, the results of one-way ANOVA showed that there is a significant difference between groups in the second four trials (average data for each mouse) (F_7,56_ = 25.92, *P*<0.001). *Post hoc* Tukey’s also showed that RSD mice receiving PBS (*P*<0.001) used more time to find the hidden platform in the target quadrant in the second four trials than their related group of control mice, indicating spatial learning impairment. While, all groups of RSD mice (Adj: *P*<0.001, M: *P*<0.001, and M+Adj: *P*<0.001) used less time, indicating spatial learning improvement. As the results showed, RSD impaired spatial learning, and multi-epitope Gag-Pol-Env-Tat derived from HIV-1 showed an improvement in spatial learning in the second four trials (Figure 1, Panel B). 

*Swimming speed-* The results of one-way ANOVA showed that there is a significant difference between groups in the first four trials (average data for each mouse) (F_7,56_ = 17.25, *P*<0.001). *Post hoc* Tukey’s also showed that RSD mice that received PBS (*P*<0.01) and Adj (*P*<0.001) had lower swimming speed in the first four trials than their related groups of control mice. Furthermore, the results of one-way ANOVA showed that there is a significant difference between groups in the second four trials (average data for each mouse) (F_7,56_ = 5.22, *P*<0.001). *Post hoc* Tukey’s also showed that RSD mice receiving Adj (*P*<0.05) had lesser swimming speed in the second four trials than their related group of control mice. As the results showed, RSD decreased swimming speed in the first four trials, and Adj groups (both control and RSD mice) showed a decrease in swimming speed; while swimming speed did not change in mice receiving multi-epitope Gag-Pol-Env-Tat derived from HIV-1 without and with Adj (Figure 1, Panel C).

***Results of spatial memory retrieval***

**Figure 2:**
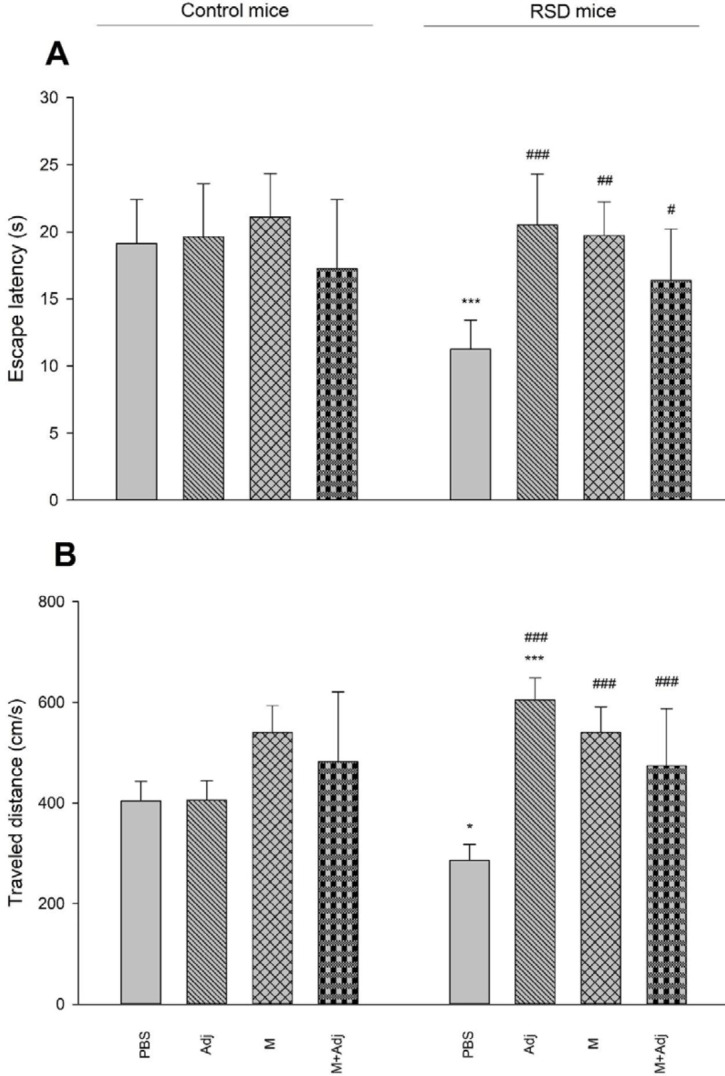
Effect of REM sleep deprivation (RSD), adjuvant montanide (Adj), multi-epitope Gag-Pol-Env-Tat derived from HIV-1 (M), and multi-epitope Gag-Pol-Env-Tat derived from HIV-1 + adjuvant montanide (M+Adj) on spatial memory retrieval (PBS as control). Panel A shows the time spent to find the hidden platform and Panel B shows the traveled distance to find the hidden platform. (***P<0.001, **P<0.01, and *P<0.05 in comparison with the related group of control mice, ###*P*<0.001, ##*P*<0.01, and #*P*<0.05 in comparison with RSD mice receiving PBS; n = 8)

*Escape latency-* The results of one-way ANOVA showed that there is a significant difference between groups (F_7,56_ = 6.31, *P*<0.001). *Post hoc* Tukey’s also showed that RSD mice receiving PBS (*P*<0.001) spent less time in the target quadrant than their related group of control mice, indicating spatial memory retrieval impairment. Furthermore, RSD-induced memory impairment was reversed in all groups of RSD mice (Adj: *P*<0.001, M: *P*<0.01, M+Adj: *P*<0.05) (Figure 2, Panel A). Thus, multi-epitope Gag-Pol-Env-Tat derived from HIV-1 showed improved RSD-induced spatial memory impairment.

*Traveled distance-* The results of one-way ANOVA showed that there is a significant difference between groups (F_7,56_ = 14.87, *P*<0.001). *Post hoc* Tukey’s also showed that RSD mice receiving PBS (*P*<0.05) spent less time in the target quadrant than their related group of control mice, indicating spatial memory retrieval impairment. However, RSD mice receiving Adj (*P*<0.001) spent more time in the target quadrant than their related group of control mice, indicating spatial memory retrieval improvement. Furthermore, RSD-induced memory impairment was reversed in all groups of RSD mice (Adj: *P*<0.001, M: *P*<0.001, M+Adj: *P*<0.001) (Figure 2, Panel B). Thus, multi-epitope Gag-Pol-Env-Tat derived from HIV-1 showed improved RSD-induced spatial memory impairment.

Note that, the results of the visible test were not significant. Thus, other reasons including psychomotor activity, fatigue, or any unrelated motivations had no effect on memory performance.

***Results of immune factors level***

**Figure 3:**
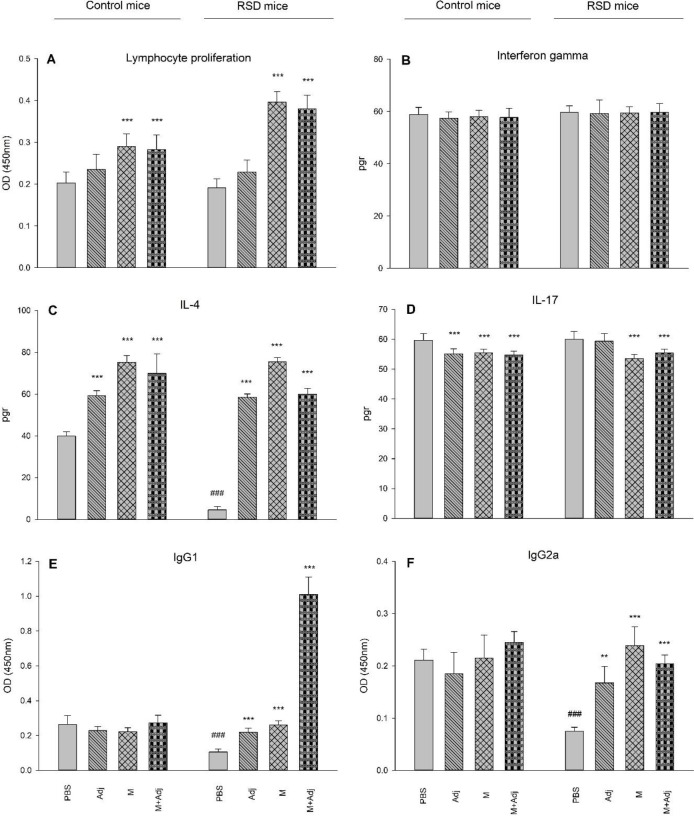
Effect of REM sleep deprivation (RSD), adjuvant montanide (Adj), multi-epitope Gag-Pol-Env-Tat derived from HIV-1 (M), and multi-epitope Gag-Pol-Env-Tat derived from HIV-1 + adjuvant montanide (M+Adj) on lymphocyte proliferation (A), interferon-gamma (B), IL-4 (C), IL-17 (D), IgG1 (E), and IgG2a (F) in mice (PBS as control). (***P<0.001, **P<0.01, and *P<0.05 in comparison with RSD mice receiving PBS, ###*P*<0.001, ##*P*<0.01, and #*P*<0.05 in comparison with the related group of control mice; n = 8)

*Lymphocyte proliferation-* The results of one-way ANOVA showed that there is a significant difference between groups (F_7,56_ = 52.86, *P*<0.001). *Post hoc* Tukey’s also showed that multi-epitope Gag-Pol-Env-Tat derived from HIV-1 with (*P*<0.001) and without (*P*<0.001) Adj increased lymphocyte proliferation in both controls (*P*<0.001) and RSD (*P*<0.001) mice (Figure 3, Panel A).

*IFN-γ-* The results of one-way ANOVA showed that there is not a significant difference between groups (F_7,56_ = 0.65, P>0.05). Thus, IFN-γ did not change in any groups (Figure 3, Panel B).

*IL-4-* The results of one-way ANOVA showed that there is a significant difference between groups (F_7,56_ = 289.65, *P*<0.001). *Post hoc* Tukey’s also showed that IL-4 was decreased in RSD mice receiving PBS (*P*<0.001) in comparison with the related group. Also, IL-4 was increased in all groups of control mice and RSD mice in comparison with their related groups (*P*<0.001) (Figure 3, Panel C). Thus, multi-epitope Gag-Pol-Env-Tat derived from HIV-1 with and without Adj increased IL-4.

*IL-17-* The results of one-way ANOVA showed that there is a significant difference between groups (F_7,56_ = 14.97, *P*<0.001). *Post hoc* Tukey’s also showed that IL-17 was decreased in all control mice in comparison with the PBS group (*P*<0.001). Also, IL-17 was decreased in all groups of RSD mice (except Adj) in comparison with the PBS group (*P*<0.001) (Figure 3, Panel D). Thus, multi-epitope Gag-Pol-Env-Tat derived from HIV-1 with and without Adj decreased IL-17.

*IgG1-* The results of one-way ANOVA showed that there is a significant difference between groups (F_7,56_ = 298.67, *P*<0.001). *Post hoc* Tukey’s also showed that IgG1 was decreased in RSD mice receiving PBS (*P*<0.001) in comparison with the related group. Also, IgG1 was increased in all groups of RSD mice in comparison with the PBS group (Adj: *P*<0.01, M and M+Adj: *P*<0.001) (Figure 3, Panel E). Thus, multi-epitope Gag-Pol-Env-Tat derived from HIV-1 with and without Adj increased IgG1 in RSD mice and reversed the effect of RSD.

*IgG2a-* The results of one-way ANOVA showed that there is a significant difference between groups (F_7,56_ = 26.11, *P*<0.001). *Post hoc* Tukey’s also showed that IgG2a was decreased in RSD mice receiving PBS (*P*<0.001) in comparison with the related group. Also, IgG1 was increased in all groups of RSD mice in comparison with the related PBS group (Adj: *P*<0.01, M and M+Adj: *P*<0.001) (Figure 3, Panel F). Thus, multi-epitope Gag-Pol-Env-Tat derived from HIV-1 with and without Adj increased IgG1 in RSD mice and reversed the effect of RSD.

## Discussion

***RSD impaired spatial memory and multi-epitope Gag-Pol-Env-Tat reversed this effect***

As the results showed, RSD impaired spatial memory, while injection of multi-epitope Gag-Pol-Env-Tat reversed this effect. Also, RSD impaired spatial learning especially in section 2, because swimming speed did not change in RSD mice in the training, while injection of multi-epitope Gag-Pol-Env-Tat reversed this effect. Note that, the impairment effect of RSD on spatial learning was not related to swimming speed, because swimming speed did not alter in RSD mice (section 2). Also, the improvement effect of multi-epitope on spatial learning was not related to swimming speed, because the swimming speed of mice treated with multi-epitope did not alter (section 2). Normal swimming speed proved that spatial learning performance was not affected by locomotor activity in these groups.

RSD impairs spatial learning and memory via attenuating hippocampal long-term potentiation (LTP) (27). LTP is an important mechanism involved in regulating memory processes and positively modulates synaptic transmission in the hippocampus (28). Also, RSD or SD can change the level of important signaling molecules involved in synaptic plasticity. For example, SD prevents hippocampal cAMP-PKA signaling and disrupts some forms of LTP in the hippocampus (29). Furthermore, during RSD or TSD, synaptic plasticity is inhibited by reducing protein kinase A (PKA) activity and phosphorylation of cAMP response element binding (CREB) protein in the hippocampus (30). Note that, CREB is involved in the synthesis of proteins involved in long-term synaptic plasticity and memory (30). SD also attenuates glutamate release in the hippocampus, while activation of N-Methyl-D-aspartate (NMDA) receptors is crucial for the induction of LTP in the hippocampus (31). Furthermore, sleep promotes neurogenesis; while, SD negatively affects proliferation and survival of newly born neuronal cells in the hippocampus and impairs memory (32), although the role of SD in modulating neurogenesis is inconsistent (33).

Brain cytokines are known as the main modulators of complex physiological and behavioral processes including sleep (34). Cytokines are so numerous; however, they can be functionally divided into two main groups: pro-inflammatory and anti-inflammatory. Pro-inflammatory cytokines are produced by activated immune cells such as microglia and have a role in the induction of amplification of inflammatory reactions (9). These cytokines are IL-1β, IL-1, IL-6, TNF-α, and IL-17 (35). It has been revealed that IL-17-producing T cells constitute a separate T-cell subset, named Th-17, which is distinct from Th-1 (pro-inflammatory) and Th-2 (anti-inflammatory) cells and has a significant role in the pathogenesis of serious autoimmune diseases such as rheumatoid arthritis (36). RSD significantly increases IL-1α, IL-1β, IL-6, IL-17, and TNF-α in rats (9). In general, SD modulates inflammatory markers in the periphery and induces negative effects on cognitive functions including memory via microglial activation-induced modulation of inflammatory cytokines (37). Inflammatory markers are significantly related to RSD-induced inhibitory avoidance memory impairment in rats (38). In addition, maternal sleep-deprived rats show poor spatial performance in the Morris water maze apparatus, due to attenuated hippocampal neurogenesis and increased IL-1β, IL-6, and TNF-α levels (39). It has been suggested that dysregulation in microglial pro- and anti-inflammatory activation has a critical role in maternal SD-induced inhibition of neurogenesis and disruption of spatial memory (39).

Note that, there is no published study investigating the effect of multi-epitope derived from HIV-1 on memory performance in rodents. This is the first study in this area and we cannot explain the behavioral results based on previous findings. For this reason, we evaluated the level of the immune factors, and now, discuss the effect of multi-epitope Gag-Pol-Env-Tat on spatial memory via analyzing the results of immune factors. 

***Effects of RSD and multi-epitope Gag-Pol-Env-Tat on immune factors level***

 As the results showed, multi-epitope Gag-Pol-Env-Tat increased lymphocyte proliferation in both control and RSD mice. Evaluating lymphocyte proliferation is an important factor to assess the quality of immune response. Proliferation (or expansion) is one of the stages crucial for the formation of long-lasting antigen-specific memory cells (40). An increase in lymphocyte proliferation following injection of multi-epitope derived from HIV-1 justifies a sufficient immune response. Our results also showed that RSD decreased IL-4, IgG1, and IgG2a levels; while, multi-epitope Gag-Pol-Env-Tat reversed these effects. Injection of multi-epitope Gag-Pol-Env-Tat also decreased IL-17 and increased lymphocyte proliferation. Microglia are the immunosensors of the stress response (including RSD) and housekeeper of immune responses, which secrete a battery of pro- and anti-inflammatory cytokines (41). Classical M1 microglia are induced following neurotoxic processes and produce high levels of pro-inflammatory cytokines such as TNFα, IL-6, IL-17, and IL-1β, while M2 microglia are induced by anti-inflammatory cytokines such as IL-4 and IL-10 (42). It has been shown that RSD can decrease IL-4 levels, leading to a decrease in the latency to paw withdrawal in pain perception (43). Maternal SD decreases the level of IL-4 and significantly elevates inflammatory cytokines in the hippocampus, leading to spatial memory deficit (44). As we know, inflammation has a negative impact on memory (45). Also, an increase in pro-inflammatory cytokines induces neuronal loss (45). Our results showed that RSD decreased IL-4 level, and HIV-1-derived multi-epitope reversed this effect. Thus, we can suggest that multi-epitope can prevent RSD-induced neuroinflammation and possible neuronal death in the hippocampus, and improve spatial learning and memory. 

Also, RSD increases the IL-17 level, which leads to induction of inflammation (9). In the present study, RSD did not change the IL-17 level. However, it could not decrease the IL-17 level, while HIV-1-derived multi-epitope decreased it in both control and RSD mice. Excess IL-17 is significantly related to different types of inflammation (46). Sleep disturbances can increase some inflammatory processes via elevating pro-inflammatory cytokines including IL-17 (47). Evidence shows a relationship between the severity of inflammatory diseases and IL-17 concentration (48). Also, there is a possible link between IL-17 level and the inflammatory response induced by RSD (9). Thus, we can suggest that multi-epitope derived from HIV-1 can reduce IL-17 and improve spatial learning and memory. 

As we know, IgG is the most common type of antibody found in blood circulation. IgG antibodies are produced following class switching and maturation of the antibody response (49). IgG antibodies predominantly participate in the secondary immune response (49). IgG has four subclasses including IgG1, IgG2, IgG3, and IgG4. IgG1 has the highest abundance in serum (49). Antibody responses to soluble protein antigens and membrane proteins primarily induce IgG1 (50). While, IgG-antibody responses to bacterial capsular polysaccharide antigens are significantly related to IgG2, and IgG2 deficiency induces the virtual absence of IgG anti-carbohydrate antibodies; although these responses are compensable by elevated levels of other IgG subclasses, especially IgG1 (51, 52). IgG2 can exist in three dominant forms based on its disulfide configuration: IgG2a, IgG2b, and IgG2a/b (53). IgG2a is superior to IgG1 for activation of complement in mice. Twenty times higher doses of IgG1, in relationship to IgG2a autoantibodies, are required to induce autoantibody-mediated pathology (54). Also, in rodents, IgG1 and IgG2a are controlled by Th-2 cytokines which induce anti-inflammatory response (55), while IgG2b and IgG2c are controlled by Th-1 cytokines via producing IFN-γ (56). Th-2 cytokines are most heavily reliant on IL-4 (57), and in the present study, all (IgG1, IgG2a, and IL-4) were increased following injection of multi-epitope. Note that, there are not enough studies investigating the role of IgGs in memory performance in mice. However, previous research has shown that anti-Aβ42 IgG1 and IgG2a are increased following co-immunization with DNA and protein mixture, leading to spatial memory improvement in mice (58). Although, some studies have shown that IgG levels do not change following spatial memory improvement/impairment (59, 60). To better understand this issue, further detailed studies are needed.

## Conclusion

Our results showed that RSD impaired spatial memory and injection of multi-epitope Gag-Pol-Env-Tat derived from HIV-1 reversed this effect. RSD decreased IL-4, IgG1, and IgG2a levels, which participate in Th-2 cytokine responses (anti-inflammatory action), while multi-epitope Gag-Pol-Env-Tat derived from HIV-1 reversed these effects. HIV-1 derived multi-epitope also increased lymphocyte proliferation and decreased IL-17 levels in both control and RSD mice. 

## Authors’ Contributions

RL Collected animal data and conducted the experiments. SV Wrote the manuscript and managed the literature search. MN Analyzed the data. FR Designed the study. All authors have approved the final version.

## Funding Information

This study was financially supported by Tehran Medical Sciences, Islamic Azad University, Tehran, Iran.

## Ethical Approval

All procedures and protocols used in the study were in accordance with the Institute for Cognitive Science Studies (ICSS) guidelines and with the National Institutes of Health Guide for the Care and Use of Laboratory Animals.

## Conflicts of Interest

The authors declare that they have no conflicts of interest.
